# Therapeutic modulation of the CD47-SIRPα axis in the pediatric tumor microenvironment: working up an appetite

**DOI:** 10.20517/cdr.2020.12

**Published:** 2020-05-11

**Authors:** Ajay Gupta, Cenny Taslim, Brian P. Tullius, Timothy P. Cripe

**Affiliations:** ^1^Division of Hematology, Oncology, Blood and Marrow Transplant, Nationwide Children’s Hospital, Columbus, OH 43205, USA.; ^2^Center for Childhood Cancer and Blood Diseases, Nationwide Children’s Hospital, Columbus, OH 43205, USA.

**Keywords:** CD47, SIRPα, immunotherapy, tumor microenvironment, pediatric cancer, innate immune system, checkpoint inhibitor, phagocytosis

## Abstract

Evasion of immune surveillance is one of the hallmarks of cancer. Although the adaptive immune system has been targeted via checkpoint inhibition, many patients do not sustain durable remissions due to the heterogeneity of the tumor microenvironment, so additional strategies are needed. The innate immune system has its own set of checkpoints, and tumors have co-opted this system by expressing surface receptors that inhibit phagocytosis. One of these receptors, CD47, also known as the “don’t eat me” signal, has been found to be overexpressed by most cancer histologies and has been successfully targeted by antibodies blocking the receptor or its ligand, signal regulatory protein α (SIRPα). By enabling phagocytosis via antigen-presenting cells, interruption of CD47-SIRPα binding leads to earlier downstream activation of the adaptive immune system. Recent and ongoing clinical trials are demonstrating the safety and efficacy of CD47 blockade in combination with monoclonal antibodies, chemotherapy, or checkpoint inhibitors for adult cancer histologies. The aim of this review is to highlight the current literature and research on CD47, provide an impetus for investigation of its blockade in pediatric cancer histologies, and provide a rationale for new combination therapies in these patients.

## Introduction

Despite the recent successes of adaptive immunotherapy, a proportion of patients have not benefitted from durable remissions due to therapeutic resistance. The role of the innate immune system checkpoint blockade is only now being recognized. The combination of innate and adaptive immunotherapy has the potential to overcome known resistance mechanisms in cancer, such as CD47 overexpression.

CD47 is an immunoglobulin-like transmembrane protein displayed on the surface of nearly all normal, healthy cells in the body as a “don’t eat me” signal to phagocytic cells. Conversely, aged or damaged cells and tumor cells often express the pro-phagocytic “eat me” ligands phosphatidylserine and calreticulin, with the balance of these opposing forces determining the activity of directly engaged phagocytic cells. The system is redundant, and a similar axis has been found between major histocompatibility complex class I expression on tumor cells and the inhibitory receptor leukocyte immunoglobulin-like receptor B1 mediating macrophage phagocytosis^[1]^. Moreover, cancer cells can release a mutated calreticulin that functions as an immunosuppressive ligand^[2]^. As with most mechanisms of immunosuppression utilized for healthy homeostasis, cancer cells often co-opt CD47 overexpression to escape innate immune surveillance by counteracting these signals^[3]^. CD47 binds to a myeloid and neuronal cell receptor called signal regulatory protein α (SIRPα), which initiates a signaling cascade within the bound phagocyte via immunoreceptor tyrosine-based inhibition motifs to inhibit immunoglobulin- or complement-induced efferocytosis of the tumor cell^[3,4]^.

The innate immune system is heavily influenced by modulation of CD47. *In vitro* studies have shown that the M1 (antitumor, inflammatory) macrophage’s ability to ingest tumor cells is altered in a CD47-dependent manner; the same has not been found to be true for M2 (pro-tumor, immunosuppressive) macrophages, perhaps indicating the evolution of CD47 overexpression by cancer to evade the macrophages trying to attack it^[5,6]^. However, in the pro-tumoral niche, CD47 appears to have a symbiotic relationship with M2 macrophages. M2-conditioned medium induces CD47 expression in cancer cells, and M2 macrophages express more SIRPα and migrate to CD47+ cells faster, while CD47+ cancer cells invade more quickly in the presence of M2 macrophages^[7]^. Dendritic cells (DC) express increased SIRPα in cancer, inducing immune tolerance, decreasing DC survival and activation, and suppressing the cytotoxic T cell response^[8]^. Natural killer (NK) cells and neutrophils have also been shown to be affected by CD47 alteration^[9-12]^. We are beginning to understand the epigenetic mechanisms as well, and CD47 activation in disease appears to turn on ubiquitin-like anti-apoptotic proteins, turn off tumor suppressor p16^[13]^, and affect targets associated with DNA methylation and histone modification^[14]^. As a therapeutic strategy, efforts are underway to block CD47-SIRPα binding and increase the innate immune recognition and phagocytosis of tumor cells. This blockade may subsequently lead to antigen presentation and adaptive T cell activation, which might then elicit further tumor destruction^[15,16]^.

CD47’s biological role independent of direct binding to SIRPα is complex, and there is evidence that it can signal on its own or through independent ligands. The ligand also interacts with thrombospondin-1 (TSP-1), which can directly regulate angiogenesis, nitric oxide signaling, T cells, and cancer stem cell renewal^[17,18]^. When interacting with αvβ3 integrins, it modulates cell adhesion, phagocytosis, and migration^[19]^. It is also known to directly affect neural migration, axon extension, and T cell co-stimulation^[16]^. In fact, CD47 blockade accentuates T cell-based immunotherapies^[20]^. Because CD47 has roles independent of SIRPα, investigators have successfully used the blockade of CD47 to affect additional interactions, including opsonization of tumor cells for antibody-dependent cellular cytotoxicity (ADCC) by the Fc receptor for IgG (FcγR) on macrophages, neutrophils, and non-SIRPα expressing NK cells^[17]^.

## Evidence in patients

CD47 was first discovered on ovarian cancer cells as an overexpressed cell surface marker^[21]^. It is now known to be overexpressed on every tumor histology tested, including ovarian, breast, colon, bladder, and prostate cancers and glioblastoma, hepatocellular carcinoma, squamous cell carcinoma, and leukemias as well^[4,16,22]^. It may be especially well-expressed on cancer stem cells^[23-25]^. In adults, high tumor CD47 expression correlates with poor progression-free and overall survival in cancer patients, including adult patients with acute lymphoblastic leukemia (ALL), acute myeloid leukemia (AML), non-Hodgkin’s lymphoma, Sézary syndrome, ovarian cancer, breast cancer, squamous cell carcinomas, gliomas, and astrocytomas^[4,5,26-31]^. Furthermore, poor response to chemotherapy (e.g., trastuzumab in breast cancer patients) may correlate with tumor cell CD47 expression^[32]^.

The data in pediatric cancers are sparse. CD47 expression was found to be an independent prognostic marker in children with ALL^[29]^. In support of this finding, anti-CD47 antibodies enhanced ALL phagocytosis *in vitro* and prevented ALL engraftment in a xenograft mouse model^[29]^. In pediatric AML, investigators found a relationship between SIRPα expression and AML FAB subtype or blast maturity, with the highest expression in the M4/M5 subsets; however, this did not correlate with outcome, and CD47 expression was uniform across samples^[33]^. In patients with osteosarcoma, increased CD47 mRNA expression and protein levels were found in tumor samples compared with paired normal tissue, which correlated with decreased progression-free and overall survival^[34,35]^. In support of this clinical observation, CD47 blockade appeared to decrease *in vivo* pulmonary metastatic formation in mouse xenograft models and increase tumor-associated macrophage (TAM) phagocytosis of osteosarcoma cells. In rhabdomyosarcoma, tissue samples for both alveolar and embryonal histologies showed high expression of CD47 and calreticulin^[36]^. Neuroblastoma patient samples were shown to have ubiquitous expression of CD47 and mouse xenograft models have demonstrated significant response to the blockade of CD47 and TSP-1^[37]^. In childhood medulloblastoma tissue samples with leptomeningeal dissemination, researchers found decreased microRNA 192 (miR-192); when they overexpressed miR-192 *in vitro*, they found that CD47 was repressed, affecting integrin alpha V activation and cell proliferation^[38]^. Finally, a variety of pediatric solid tumor histologies, including Ewing sarcoma, medulloblastoma, atypical teratoid/rhabdoid tumor, primitive neuroectodermal tumor, pediatric high-grade glioma, and diffuse intrinsic pontine glioma were found to have diffuse CD47 expression; the brain tumors all showed response to CD47 blockade in xenograft models^[39,40]^.

**Figure 1:**
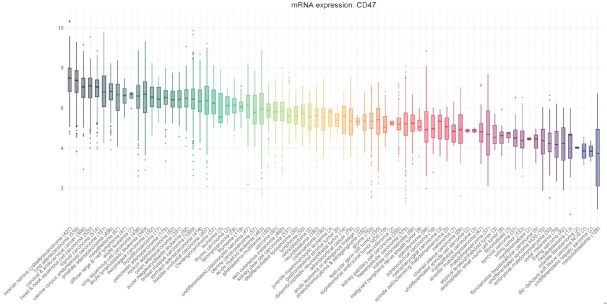
CD47 expression across all cases. We included all Treehouse data with at least 2 samples and ordered them by their average normalized expression. Y-axis represents log2 normalized or log2(TPM + 1) expression of CD47. X-axis shows the diseases with the number of samples in the parenthesis

To help guide studies of CD47 blockade in pediatric oncology, we sought to identify which histologies express high levels of CD47. We analyzed publicly available RNA-seq expression data from the Treehouse Childhood Cancer Initiative at the UC Santa Cruz Genomics Institute, which includes a total of 12,211 samples of both adult and pediatric cases [Figure 1]. We downloaded RNA-seq expression data and their associated patient-privacy protected clinical data (https://treehousegenomics.soe.ucsc.edu/public-data/#tumor_v10_polyA) on December 4, 2019. All expression data were uniformly processed and normalized by Treehouse Childhood Cancer Initiative (https://github.com/BD2KGenomics/toil-rnaseq). Gene expression was quantified as transcript per million (TPM). R packages data.table 1.12.2, ggplot2 3.2.1 and R 3.6.0 were used to plot the CD47 expression panel^[41-43]^.

**Figure 2:**
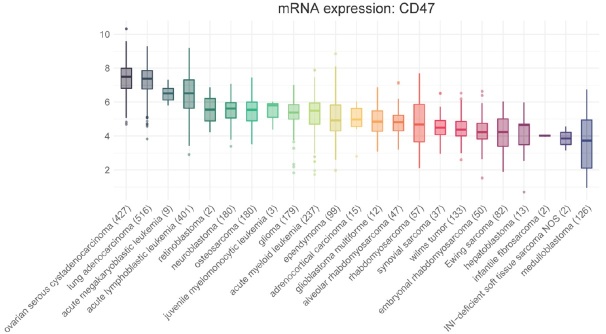
CD47 across pediatric histologies. For comparison, we included the top two adult expressing tumor types (ovarian serous cystadenocarcinoma and lung adenocarcinoma). For those histologies that are shared between pediatric and adults (e.g., acute lymphoblastic leukemia, acute megakaryoblastic leukemia, acute myeloid leukemia, adrenocortical carcinoma, glioblastoma multiforme, and glioma), we only included cases under age 19; for those histologies that are classically a pediatric diagnosis (all others), we included all ages to capture data in young adults with pediatric diagnoses as well. We only included histologies with at least 2 patient samples. We ordered the data by their average normalized expression. Y-axis represents log2 normalized or log2(TPM + 1) expression of CD47.. X-axis shows the diseases with the number of observations in parentheses

We also created a “pediatric cancer” data subset, which included all ages for histologies that are classically diagnosed in pediatric, adolescent or young adult patients but was limited to those patients under 19 years for histologies that span a broad age range [Figure 2]. Data are shown normalized to the expression of all genes across the database.

On average, essentially all cancers express CD47 mRNA, mostly ranging 2-8 log2 (4-256 TPM for all genes). Among pediatric cancers, we find the highest expression of CD47 in M7 AML and ALL, nearly as high as the highest adult cancers. Expression is slightly lower in a variety of pediatric solid tumors such as retinoblastoma, neuroblastoma, osteosarcoma and others, with medulloblastoma showing the lowest average expression. The rank order is somewhat reminiscent of tumor mutational burden^[44]^, suggesting there may be a correlation between tumor immunogenicity and CD47 expression. That said, the range of expression amongst samples within each histology is quite wide. Thus, as the blockade of CD47 depends on its expression, it will likely vary considerably from case to case.

**Figure 3:**
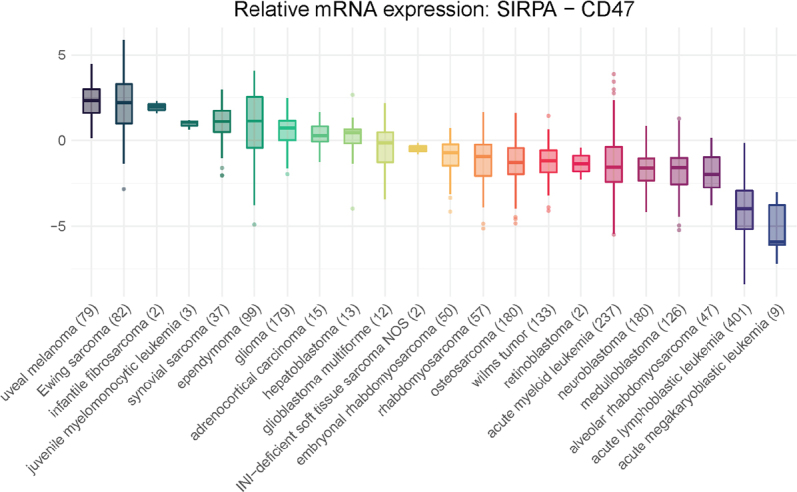
SIRPα to CD47 relative mRNA expression. Included are the expression panels for pediatric cases as described in Figure 2. Y-axis in these two panels is the relative expression of SIRPα as compared to CD47 expression [i.e., log2(TPM + 1) expression of SIRPα - log2(TPM + 1) expression of CD47]. Positive value means expression of SIRPα is log2 fold change higher than CD47 expression and vice versa. Boxplots are ordered by their average relative expression. SIRPα: signal regulatory protein α

Given the suggested importance of surface expression of corresponding ligands on tumor immune infiltrate, we also examined the same pediatric cancers for SIRPα [Figure 3]. Many histologies demonstrate surface expression of SIRPα on par with that of CD47, suggesting that the interaction between the two is likely relevant. The lower expression of SIRPα in leukemias is likely because many of the samples were taken from peripheral blood and thus do not reflect the bone marrow microenvironment. We also studied additional ligands known to interact with CD47 such as TSP-1 and signaling lymphocytic activation molecule F7 (SLAMF7) (data not shown). Our data demonstrate a similar relationship between TSP-1 and CD47, with a majority of pediatric solid tumor microenvironments showing equivalent expression of both ligands. As mentioned earlier, TSP-1 is another ligand for CD47 on many cell types, including innate immune cells, and studies have shown that CD47 binding to TSP-1 affects macrophage recruitment, IL-1β production, and the expression of cancer stem cell transcription factors^[45]^. Increased SLAMF7 expression on either tumor or immune cells may govern a macrophage’s ability to engulf hematopoietic tumor cells in response to CD47 blockade^[46]^, although this result has been called into question^[47]^. Our data seem to echo the questionable role of this ligand, and SLAMF7 shows uniformly low expression compared to CD47.

## Therapeutic advances/combination therapies

The observed success of immunotherapy in today’s therapeutic landscape indicates that, while we have established an anticancer modality that can be effective, significant improvements are needed to broaden survival benefit over time. Although CD47 has a major role in regulating phagocytosis, it is actually FcγR engagement that is requisite for phagocytosis; mice that are CD47-deficient have a largely normal phenotype, other than mild anemia or thrombocytopenia, without overt autoimmunity^[17]^. CD47 blockade may also trigger T cells via stimulator of interferon genes (STING)-based cytosolic sensing of tumor cell DNA^[20]^. Tenascin C (an extracellular matrix protein) and hypoxia-inducible factor are also thought to mediate CD47-associated changes in the tumor microenvironment^[24,48]^.

On the other side of the interaction, SIRPα blockade may achieve similar goals via antibody-mediated tumor cell destruction, increased licensing of the cytokine IL-12, and negative regulation of pro-inflammatory pathways^[8,32]^. IL-12 mediates T helper type 1 cell (Th1) polarization of activated CD4 T cells and subsequent amplification of the CD8 cytotoxic lymphocyte response^[8]^. When mice are administered an antigen-pulsed DC-based vaccine with lentiviral expression of miRNA that silences SIRPα, there is greater DC activation, T cell proliferation, interferon gamma production, and cytolytic activity^[8]^. Anti-SIRPα antibodies appear to target neutrophils and macrophages that are contributing to tumor growth *in vitro* and *in vivo*^[49]^. Some macrophages express both SIRPα and CD47, and it was recently shown that inhibiting both receptors on the same macrophage creates a hyper-phagocytic state^[50]^. The effect of blocking SIRPα has been shown to be attenuated by the depletion of macrophages, CD8+ T cells, and NK cells^[51]^. Moreover, CD47 overexpression may blunt the therapeutic action of monoclonal antibodies, and therefore, CD47 blockade would enhance antibody efficacy^[52]^. Additional strategies to block this axis involve engineered SIRPα monomers or exosomes with SIRPα that have a high affinity for CD47 and that would similarly lower the macrophage threshold for phagocytosis and, as a result, T cell activation^[15,53]^. A comprehensive review of the various types of anti-CD47 and anti-SIRPα blocking agents has recently been published^[46]^.

On the basis of the findings reported so far, it is logical to hypothesize that solely blocking the CD47-SIRPα axis in humans will be insufficient to elicit an antitumor phagocytic effect. Thus, combination therapies and the identification of new checkpoints to inhibit, especially from both the adaptive and innate immune standpoints, may help address this deficit^[54]^. Investigators have already examined various *in vitro* and *in vivo* combinations with anti-CD47, including PD-L1 or CTLA-4 blockade, monoclonal antibodies, chemotherapy, and radiation. There is evidence that TAMs express PD-1, have increased PD-1 expression over time and with higher disease stage, and have a decreased ability to phagocytose PD-L1-expressing tumor cells^[55]^, lending a rational approach to combination blockade of PD-L1 and CD47^[52]^. Similar evidence has been presented for CTLA-4^[56]^. Researchers designed epithelial cell adhesion molecule-targeted cationic liposomes containing siRNA for both PD-L1 and CD47 and found significant *in vivo* decrement in solid tumor burden and metastases^[57]^. By combining CD47-SIRPα disruption with IgA antibodies against HER2, one group was able to enhance tumor cell opsonization and decrease tumor burden via neutrophil trogocytosis, a method of acquiring target cell plasma membrane fragments^[10]^. Similar results were produced with the addition of monoclonal antibodies such as rituximab (anti-CD20), alemtuzumab (anti-CD52), lorvotuzumab (anti-CD56), trastuzumab (anti-HER2), cetuximab (anti-EGFR), and anti-CD271 (nerve growth factor receptor)^[53,58,59]^. Synergy has been demonstrated between CD47 binding and anti-angiogenic, anti-T cell receptor mimetic for PRAME (preferentially expressed antigen in melanoma), tyrosine kinase inhibitor (sorafenib), or anti-Bcl2 (venetoclax) therapy^[60-63]^. When considering the combination of chemotherapy with CD47 blockade, chemotherapy may lead to increased tumor infiltration by antigen-presenting cells (APCs), increased antigen release, and increased calreticulin expression^[64,65]^, but may also suppress the immune system and thus blunt the effect of CD47 blockade^[20]^. It may be that the sequence of therapies will be important. For example, the *in vivo* combination of anti-CD47 treatment with cyclophosphamide or paclitaxel for mouse A20 lymphoma tumors resulted in maximum synergy with chemotherapy given 1 day prior to CD47 blockade rather than 3 days after^[20]^. Chemotherapy may induce the infiltration of TAMs into the tumor, and anti-CD47 therapy could subsequently convert them into effector cells^[66]^. Anthracyclines can mediate susceptibility to a blocking antibody against CD47, increase translocation of calreticulin to the cell surface, and intensify macrophage activity^[36,67]^; *in vivo* studies have shown successful combination therapy against osteosarcoma^[68]^. In this setting, CD47 blockade may have cardioprotective properties mediated by an increase in autophagy^[69]^. Finally, a particularly innovative approach using an oxaliplatin prodrug and a pegylated photosensitizer activated by tumor microenvironment-associated matrix metalloproteinase-2 (MMP2) showed synergy with injection of CD47 antibodies into the tumor^[70]^.

Local control may be aided by anti-CD47 treatment. In mice treated with debulking surgery for glioblastoma multiforme xenografts, antibodies injected into the resection cavity led to prolonged survival, increased macrophage infiltration, and increased pro-inflammatory cytokines^[71]^. Near-infrared photoimmunotherapy has been developed with CD47 antibodies with good local tumor control *in vivo*^[72]^. CD47 blockade may also enhance tumor radiosensitivity via improved CD8 T cell immunosurveillance in syngeneic mouse models^[18]^, STING-based tumor visibility^[73]^, and selective upregulation of protective pathways against oxidative stress and upregulation of DNA repair in normal tissues^[74]^. Signals for autophagy are turned on in endothelial cells and T cells, resulting in increased blood flow within tumors and enhancing the penetration of cytotoxic lymphocytes both locally and possibly at distant tumor sites^[18]^, the off-stage, on-target result known as the abscopal effect. Treatment with anti-CD47 antibody plus anti-HER2 antibody or temozolamide in mice results in radiosensitization and improves survival over that with either therapy alone^[73,75]^. In another study, microRNA 222 (miR-222) was found to negatively regulate CD47 expression, and overexpression of miR-222 enhanced cancer cell radiosensitivity via the CD47-pERK pathway^[76]^. While signals for autophagy may be radioprotective for normal tissue, other studies have shown that blocking tumor autophagy with chloroquine and anti-CD47 is an effective antitumor strategy *in vivo*^[77]^.

In clinical trials, investigators have started adding anti-CD47 therapy to well-established lines of therapy in different adult cancers, including a successful study in rituximab-resistant non-Hodgkin lymphoma with a humanized anti-CD47 antibody, Hu5F9-G4, and rituximab^[78]^. Preclinical work on this synergy appeared to support two mechanisms for its action, namely Fc receptor (FcR)-independent anti-CD47 blockade and FcR-dependent pro-phagocytosis signal via rituximab^[27]^. In addition, rituximab induces complement and NK-mediated ADCC^[78]^. In that study, 22 refractory non-Hodgkin’s lymphoma patients were treated in a phase 1b study of Hu5F9-G4 plus rituximab. It was very well tolerated with two grade 3-4 hematological adverse events and an impressive objective response rate of 50%, with 36% of the patients having a complete response. The median duration of response was not reached at more than 6 to 8 months of follow-up^[78]^. Prior concerns of the ubiquity of CD47 on normal hematopoietic cells that may act as an “antigen sink” with subsequent off-target toxicity may be mitigated by these results.

On-going trials are utilizing CD47 blockade plus PD-1/PD-L1 inhibitors (NCT02663518, NCT02890368, NCT03013218 and NCT03530683, NCT03558139), ramucirumab and paclitaxel (NCT03013218), 5-FU and cisplatin (NCT03013218), azacitidine (NCT03248479), cetuximab (NCT02953782), carfilzomib (NCT03530683), radiation (NCT02890368), pegylated interferon-α2a (NCT02890368), and talimogene laherparepvec (T-Vec) (NCT02890368). Preliminary results from NCT03248479 demonstrate good tolerance of combination therapy with azacitidine^[79]^. However, none of these trials allow patients under 18 years of age.

Next-generation CD47 blockade has resulted in bispecific antibody platforms that can also target CD19 or CD20 in a mouse lymphoma model^[80-82]^, CD33 or CD123 in AML^[83-85]^, CD40 in colon carcinoma^[86]^, tumor-associated antigens such as mesothelin^[87]^ and VEGFR1^[60]^ in non-small cell lung cancer, and even dual blockade of CD47 and SIRPα^[88,89]^ or SIRPα and PD-L1^[90]^; however, trials in humans have yet to be conducted. CD96, like CD123, has been suggested as a leukemic stem cell-specific molecule that also engages Fc receptors on phagocytes^[26]^, and might be an effective target in combination with anti-CD47. CD47 antibody has been fused with GM-CSF, enabling M1 macrophage polarization and antitumor effect^[91]^. Nanobodies (single-domain antibody fragments) targeting CD47 have been constructed with decreased affinity for human red blood cells and also conjugated to rituximab as a novel bispecific antibody with *in vivo* antitumor effect^[92]^. Chimeric antigen receptor-T (CAR-T) cells have been engineered to emit these nanobodies and may have the ability to simultaneously produce nanobodies for different targets, including CD47, PD-L1, or CTLA-4^[93]^. One group has capitalized on the cytotoxicity of certain CD47 antibodies, creating an antibody that shows both a direct antitumor effect and increased macrophage phagocytosis and decreased red blood cell destruction^[94]^. Oncolytic adenoviruses expressing a SIRPα-Fc fusion protein have been shown to have macrophage-dependent cytotoxicity against ovarian xenografts, in addition to the inherently lytic properties of the virus, and they deserve broader study^[95]^. More recently, two groups independently used syngeneic inactivated tumor cells deficient in CD47 as a vaccine *in vivo* to stimulate immune recognition of existing mouse melanoma or lymphoma^[9,96]^. The first study noted that the vaccinated mice had increased tumor-infiltrating NK cells; in the tumors that failed to respond to the vaccines, there were elevated regulatory T cells, higher PD-L1 expression, and increased M2 macrophages^[9]^, all together portraying an exhaustion phenotype. When the second group employed combination blockade of tumor vaccine and PD-L1, they found synergistic antitumor responses^[96]^.

## Conclusion

**Figure 4:**
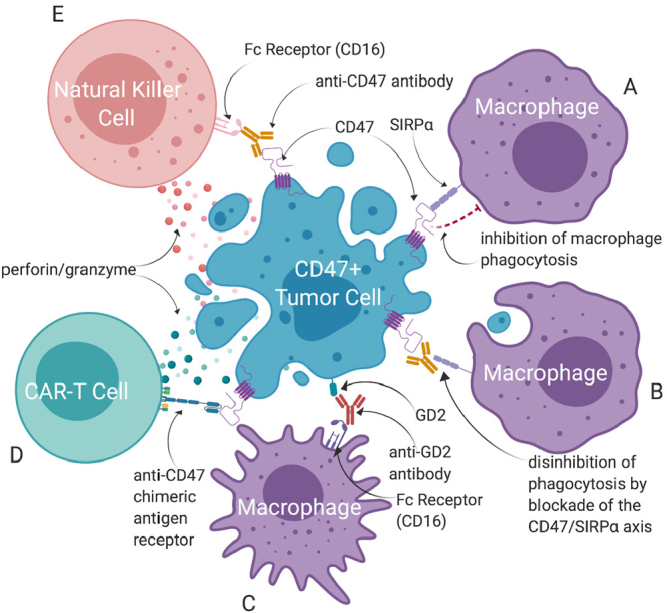
Targets to prioritize in combination with CD47 blockade. Targeting CD47 can alter immune effector response to the tumor microenvironment in a variety of ways. Where typically the interaction between SIRPα on the macrophage and CD47 on the tumor inhibits tumor cell phagocytosis by the macrophage (A), anti-CD47 antibodies can disrupt this interaction, permitting phagocytosis (B). This blockade of the CD47/SIRPα axis can be paired with other targeted antibody therapies, exemplified here with anti-GD2 antibody dinutuximab, to target tumor cells through the macrophage Fc receptor (C). T cells can be genetically engineered to express anti-CD47 CAR directly targeting CD47+ tumor cells for lysis (D). Similarly, natural killer cells can engage CD47+ tumor cells through antibody-dependent cellular cytotoxicity via anti-CD47 antibody interaction with the Fc receptor (E). CAR: chimeric antigen receptors; SIRPα: signal regulatory protein α

Advances in immunotherapy have begun to involve the long-ignored innate immune system, but pediatric cancers have yet to benefit. Engaging phagocytes allows both direct tumor kill and indirect engagement of cytotoxic T cells via APCs and STING. This dual threat is further strengthened by combination with additional immunotherapy aimed at T cells (checkpoint inhibition) or FcR and ADCC (monoclonal antibodies) and may even get to the root of treatment resistance by eliminating cancer stem cells. Future trials may prioritize the combination of anti-CD47 therapy with targeted antibodies against known receptors such as GD2 [Figure 4], CD47-specific CAR-T cells, NK cells, or tumor vaccines. According to our analysis, while CD47 blockade may be effective across many, if not all, cancer histologies, the patients most likely to benefit will be those with the highest surface expression of this marker. Given the range of variability in expression, patients should be selected on a case-by-case basis.

As we move the focus to hitherto unexplored territories, including rare and pediatric histologies and next-generation CD47 blockade in combination with more effective immunotherapies and chemotherapies, we will hopefully overwhelm cancer’s voracious appetite by endowing our immune system with its own set of teeth.

## Declarations

### Copyright

© The Author(s) 2020.
